# Clinician Perspectives on AI-Generated Drafts of Patient Test Result Explanations

**DOI:** 10.1001/jamanetworkopen.2025.28794

**Published:** 2025-08-22

**Authors:** Shreya J. Shah, Abishek Nair, Kirsten Murtagh, Stephen P. Ma, Kyle Vogt, Danyelle Clutter, Liban Sheikh, Haley Schmidt, Margaret Smith, Arun Lakhotia, Lance Bullock, Aditya Bhasin, Michael A. Pfeffer, Christopher Sharp, Steven Lin, Patricia Garcia

**Affiliations:** 1 Department of Medicine, Stanford University School of Medicine, Stanford, California; 2 Stanford Healthcare AI Applied Research Team, Division of Primary Care and Population Health, Stanford University School of Medicine, Stanford, California; 3 Technology and Digital Solutions, Stanford Medicine, Stanford, California

## Abstract

This quality improvement study evaluates clinician perspectives on the usability and utility of generative artificial intelligence (AI)–based large language model tool to draft result comments for laboratory, imaging, and pathology results.

## Introduction

The 21st Century Cures Act mandating immediate release of test results to patients enhanced transparency but also led to patient anxiety.^1,2^ Patients prefer results explained directly by clinicians.^2^ Generative artificial intelligence (AI) provides an opportunity to enhance patient understanding of test results^3^ while reducing clinician burden and burnout.^4,5^ Stanford Health Care developed a novel generative AI-based large language model (LLM) tool to draft result comments for laboratory, imaging, and pathology results. This study evaluated clinician perspectives from a pilot implementation guided by the RE-AIM/PRISM framework,^5,6^ assessing usability, utility, and suggestions for tool improvement.

## Methods

This prospective quality improvement study was conducted from January 13 through March 31, 2025, and followed the SQUIRE reporting guideline. The institutional review board (IRB) at Stanford University determined that this study met the criteria for quality improvement and was exempt from the need for IRB–mandated consent. Stanford Health Care developed an electronic health record–integrated tool for drafting result comments similar in concept to a previously studied AI inbox message replies tool.^5^ Claude 3.5 Sonnet (Anthropic) was selected as the LLM for the pilot based on its superior response time, fidelity to prompt instructions, and ability to generate outputs resembling result comments from clinicians. Primary care clinicians across both faculty and community practice networks were invited to participate in 2 waves staggered by 4 weeks, with postsurveys administered after 4 or 8 weeks of tool use. Surveys, guided by RE-AIM/PRISM, assessed usability and utility (5-point Likert scale), and perceived time impact per result comment (eMethods in Supplement 1). Descriptive statistics were used to summarize Likert responses; “strongly agree” and “agree” responses were combined to report survey results. Free-text survey responses were systematically analyzed by 2 researchers (S.J.S., K.M.) using deductive thematic coding followed by inductive theme identification during consensus reconciliation. Comments were segmented into phrases; assigned a positive, negative, or neutral sentiment; and summarized. Phrases were allowed to have multiple codes. Microsoft Excel was used for analysis.

## Results

Of 244 clinicians who used the tool at least once, 93 (38.1%) completed postsurveys (62 [66.7%] female; 31 [33.3%] male; 46 [49.5%] with ≥15 years after training in practice). Clinicians reported favorable usability (79 [84.9%] found the tool easy to use) and utility, particularly for laboratory (67 [72.0%]) and imaging (59 [63.4%]) results, improved efficiency (66 [71.0%]), and higher quality explanations (67 [72.0%]) (Figure). Over half reported using the tool frequently (54 [58.1%]) and that the tool was ready for broad implementation (50 [53.8%]). Most (77 [82.8%]) anticipated long-term use, and 39 (41.9%) felt motivated to send result comments to patients more frequently. Median perceived time savings per result was 1.1 minutes (range, 5.0 minutes saved to 3.3 minutes additional time spent).

**Figure.**
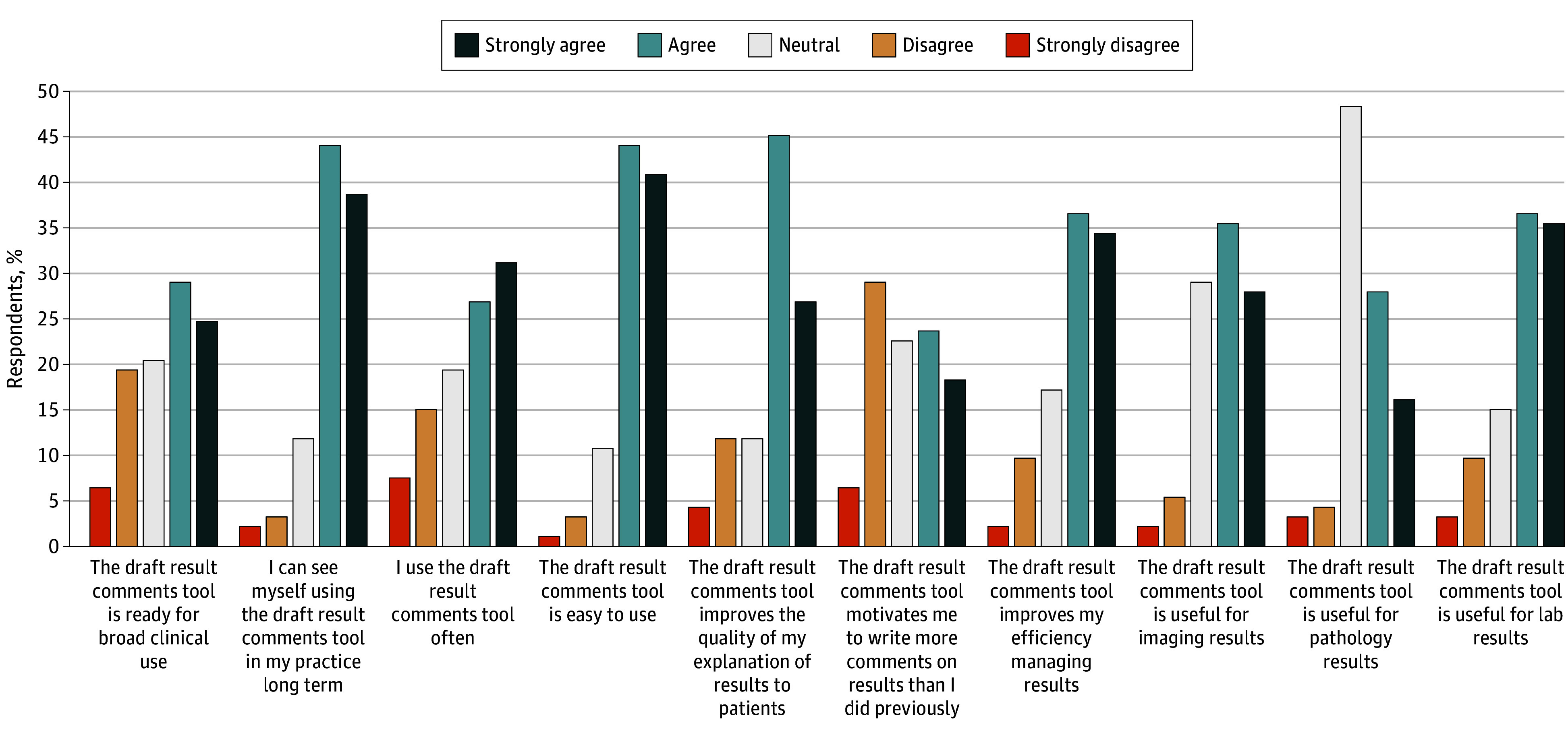
Postsurvey Likert Scale Results From 93 Respondents

Themes from free-text responses included positive sentiments regarding tool utility and patient engagement and negative sentiments related to content accuracy and completeness (Table). Clinicians offered specific suggestions for tool optimization (eg, including more patient context from recent visit notes) and improved workflow integration (eg, updating drafts sequentially for results released over time).

**Table.** Qualitative Encoding of Free-Text Comments From Surveys

Theme	Representative quotations	Comments, No.			
		Negative	Neutral	Positive	Total
Overall tool utility	Positive: “Very helpful in explaining results to patients.” Negative: “The result comments of ‘this is common for your age’” is not helpful. The order of lab results seems random and would be better if customizable.”	2	3	20	25
Content accuracy	Positive: “It is getting better and more accurate in the area of lab results and imaging results.” Negative: “Lab results needs more work—some labs not addressed or incorrectly addressed.”	13	1	2	16
Content completeness	Positive: “They touch on all results, which I often used to skip, but I imagine the patient appreciates.” Negative: “Some result comments omit providing info to components, which is important with abnormal lab values.”	6	1	4	11
Impact on workflow	Positive: “I liked how the tool pulled up prior historical data easily—that makes it a good cross reference.” Negative: “When new results arrive on a single order placement (and the Comment is recreated), it’s a clunky workflow since the other results already have comments attached.”	3	1	6	10
Draft result comment length	Positive: “Particularly useful when covering another MD’s inbox! Appreciate the ‘to the point’ brevity in human language.” Negative: “Would also like it if it can be more concise sometimes it gives a little too much information.”	6	0	1	7
Future use and readiness for scale	Positive: “I am willing to continue to use, as I’m confident the tool will improve and ultimately be a time-saver.” Negative: “May need more time and refinement before prime-time broader use of the tool.”	2	0	5	7
Impact on patient engagement	Positive: “I love the quality and content of the drafts. They are more complete and empathetic than I historically wrote.” Negative: “When I have used the AI-drafted interpretation, the patient will message me with more questions so I’ve stopped using this feature as much as I would have liked.”	1	0	6	7
Impact on time and efficiency	Positive: “I think it really helps me release results quickly to the patients who are waiting and act on them accordingly.” Negative: “I use a dotphrase for lab comments and the AI response takes longer.”	1	1	4	6
Voice and tone	Positive: “Great tool—I really like the tone of the comments as well!” Negative: “It does not sound like ‘me.’ I still edit for clarity.”	1	1	2	4
Utility specific to imaging results	Positive: “I find the radiology comments to be the most helpful and the language used sounds the most like language that I would produce myself.” Negative: NA	0	1	2	3
Utility specific to lab results	Positive: NA Negative: “I like echo, radiology report. For lab results, it does not interpret all the labs were done, missing some important labs and I’m not sure why.”	3	0	0	3
Utility specific to pathology results	Neutral: “I’m not sure whether it works well for pathology—not enough experience!”	0	2	0	2
Total	NA	38	11	53	102

Abbreviations: AI, artificial intelligence; NA, not applicable.

## Discussion

This study demonstrated the utility of a generative AI tool for drafting test result explanations, highlighting ease of use, improved efficiency, and higher-quality explanations. Barriers to adoption included content accuracy and completeness. Limitations include selection bias, limited generalizability beyond primary care, and underrepresentation of certain test types (eg, pathology). While these early results suggest that AI-generated draft result comments could help reduce clinician burden and enhance patient experience, further optimization grounded in clinician feedback is needed to improve accuracy and completeness of draft explanations. Additional improvements should focus on optimizing prompts, updating the LLM, incorporating patient-specific context, and streamlining workflow integration. Future evaluations should quantify impacts on clinician inbox burden (time spent, message volume) and consider patient perspectives.

## Notes

Supplement 1. **eMethods.** Survey

Supplement 2.
Data Sharing Statement
