# Supplementary material for: Clinician Perspectives on AI-Generated Drafts of Patient Test Result Explanations
Source: JAMA Netw Open. 2025 Aug 22;8(8):e2528794. doi: 10.1001/jamanetworkopen.2025.28794 (PMC12374212; doi:10.1001/jamanetworkopen.2025.28794)
Supplement: Supplement 1. — eMethods. Survey [file jamanetwopen-e2528794-s001.pdf]

Supplemental Online Content

Shah S, Nair A, Murtagh K, et al. Clinician perspectives on AI-generated drafts of patient test result explanations. *JAMA Netw Open.* 2025;8(8):e2528794.
doi:10.1001/jamanetworkopen.2025.28794

eMethods. Survey

This supplemental material has been provided by the authors to give readers additional information about their work.

© 2025 Shah S et al. *JAMA Network Open*.

eMethods. Survey

Start of Block: Default Question Block

Q1 Thank you for participating in the AI-generated draft result comments pilot! Please indicate to what degree you agree or disagree with the following statements: The draft result comments tool is useful for lab results

- ○ Strongly Agree (1)
- ○ Agree (2)
- ○ Neutral (3)
- ○ Disagree (4)
- ○ Strongly Disagree (5)

Q2 The draft result comments tool is useful for pathology results

- ○ Strongly Agree (1)
- ○ Agree (2)
- ○ Neutral (3)
- ○ Disagree (4)
- ○ Strongly Disagree (5)

© 2025 Shah S et al. *JAMA Network Open*.

Q3 The draft result comments tool is useful for imaging results

- Strongly Agree (1)
- Agree (2)
- Neutral (3)
- Disagree (4)
- Strongly Disagree (5)

Q4 The draft result comments tool improves my efficiency managing results

- Strongly Agree (1)
- Agree (2)
- Neutral (3)
- Disagree (4)
- Strongly Disagree (5)

Q5 The draft result comments tool motivates me to write more comments on results than I did previously

- Strongly Agree (1)
- Agree (2)
- Neutral (3)
- Disagree (4)
- Strongly Disagree (5)

© 2025 Shah S et al. *JAMA Network Open*.

Q6 The draft result comments tool improves the quality of my explanation of results to patients

- Strongly Agree (1)
- Agree (2)
- Neutral (3)
- Disagree (4)
- Strongly Disagree (5)

Q7 The draft result comments tool is easy to use

- Strongly Agree (1)
- Agree (2)
- Neutral (3)
- Disagree (4)
- Strongly Disagree (5)

Q8 I use the draft result comments tool often

- Strongly Agree (1)
- Agree (2)
- Neutral (3)
- Disagree (4)
- Strongly Disagree (5)

© 2025 Shah S et al. *JAMA Network Open*.

Q9 I can see myself using the draft result comments tool in my practice long term

- Strongly Agree (1)
- Agree (2)
- Neutral (3)
- Disagree (4)
- Strongly Disagree (5)

Q10 The draft result comments tool is ready for broad clinical use

- Strongly Agree (1)
- Agree (2)
- Neutral (3)
- Disagree (4)
- Strongly Disagree (5)

Q11 Please estimate the impact of using the draft result comments tool on the time it takes to write a single result comment (in minutes per comment)

	Time saved		No time difference		Additional time spent	
	-5	-3	-1	1	3	5
Mins ()						

© 2025 Shah S et al. *JAMA Network Open*.

Q12 How likely are you to recommend using this draft result comments tool to a friend or colleague

- ◯ 0 (0)
- ◯ 1 (1)
- ◯ 2 (2)
- ◯ 3 (3)
- ◯ 4 (4)
- ◯ 5 (5)
- ◯ 6 (6)
- ◯ 7 (7)
- ◯ 8 (8)
- ◯ 9 (9)
- ◯ 10 (10)

Q13 Please share any additional feedback you have about your experience using this draft result comments tool. Your feedback will help us better understand and determine potential long-term use of the tool

____

____

____

____

____

End of Block: Default Question Block

Start of Block: Demographics

© 2025 Shah S et al. *JAMA Network Open*.

*

Q14 Please enter your SID:

____

____

____

____

____

Q15 Please select your gender:

- ○ Male (1)
- ○ Female (2)
- ○ Non-binary (3)
- ○ Prefer not to say (4)

Q16 Please select your years after training in practice

- ○ 0-4 (1)
- ○ 5-9 (2)
- ○ 10-14 (3)
- ○ 15+ (4)
- ○ Prefer not to say (5)

End of Block: Demographics

© 2025 Shah S et al. *JAMA Network Open*.
