# Supplementary material for: Clinician Perspectives on AI-Generated Drafts of Patient Test Result Explanations
Source: JAMA Netw Open. 2025 Aug 22;8(8):e2528794. doi: 10.1001/jamanetworkopen.2025.28794 (PMC12374212; doi:10.1001/jamanetworkopen.2025.28794)
Supplement: Supplement 2. — Data Sharing Statement [file jamanetwopen-e2528794-s002.pdf]

Data Sharing Statement

Shah. Clinician Perspectives on AI-Generated Drafts of Patient Test Result Explanations. *JAMA Netw Open*. Published August 25, 2025. doi:10.1001/jamanetworkopen.2025.28794

Data
Data available: No
